# Supplementary material for: Electron transfer rules of minerals under pressure informed by machine learning
Source: Nat Commun. 2023 Mar 31;14:1815. doi: 10.1038/s41467-023-37384-1 (PMC10066309; doi:10.1038/s41467-023-37384-1)
Supplement: Supplementary file 1 — Extended data figures and tables [file 41467_2023_37384_MOESM1_ESM.pdf]

Extended data figures and tables for

Electron Transfer Rules of Minerals under Pressure Informed by Machine Learning

Yanzhang Li, Hongyu Wang, Yan Li*, Huan Ye, Yanan Zhang, Rongzhang Yin, Haoning Jia, Bingxu Hou, Changqiu Wang, Hongrui Ding, Xiangzhi Bai*, Anhuai Lu*

Correspondence to: ahlu@pku.edu.cn
liyan-pku@pku.edu.cn
jackybxz@buaa.edu.cn

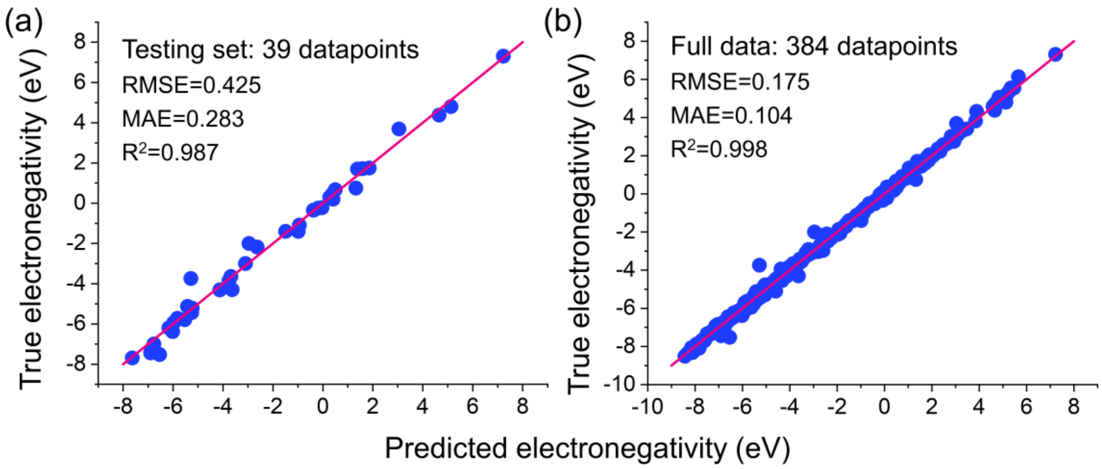

Extended Data Fig. 1 Performances of deep learning model on testing test (a) and full data (b). The squared Pearson correlation coefficient (R^2), small mean absolute error (MAE) and root-mean-square error (RMSE) of the predicted results compared with those true values are given.

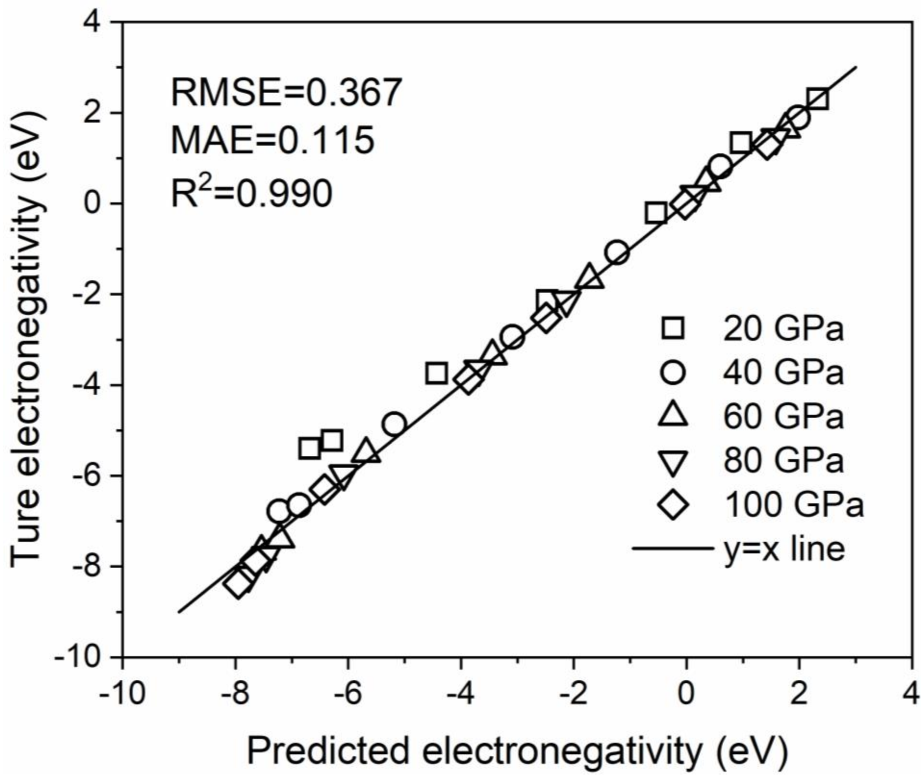

Extended Data Fig. 2 Performances of deep learning model on the testing set consisting of Na, Mg, Ni, C, N, Li and Au at 20, 40, 60, 80 and 100 GPa. The squared Pearson correlation coefficient (R^2), small mean absolute error (MAE) and root-mean-square error (RMSE) of the predicted results compared with those true values are given.

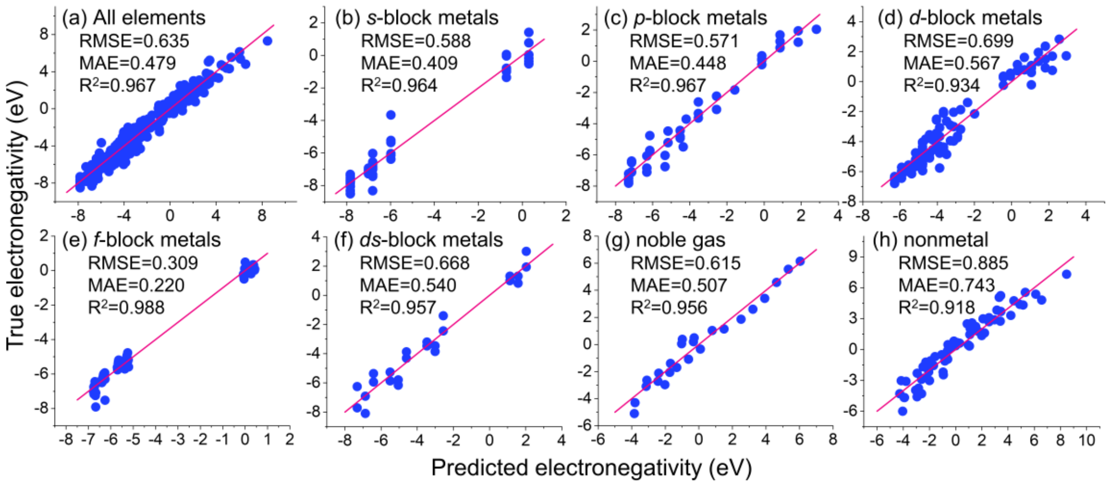

Extended Data Fig. 3 Fitting performances of empirical formulas for all elements (a) and elements in *s*-block metals (b), *p*-block metals (c), *d*-block metals (d), *f*-block metals (e), *ds*-block metals (f), noble gas (g), and nonmetal (h). The squared Pearson correlation coefficient (R^2), small mean absolute error (MAE) and root-mean-square error (RMSE) of the predicted results compared with those true values are given.

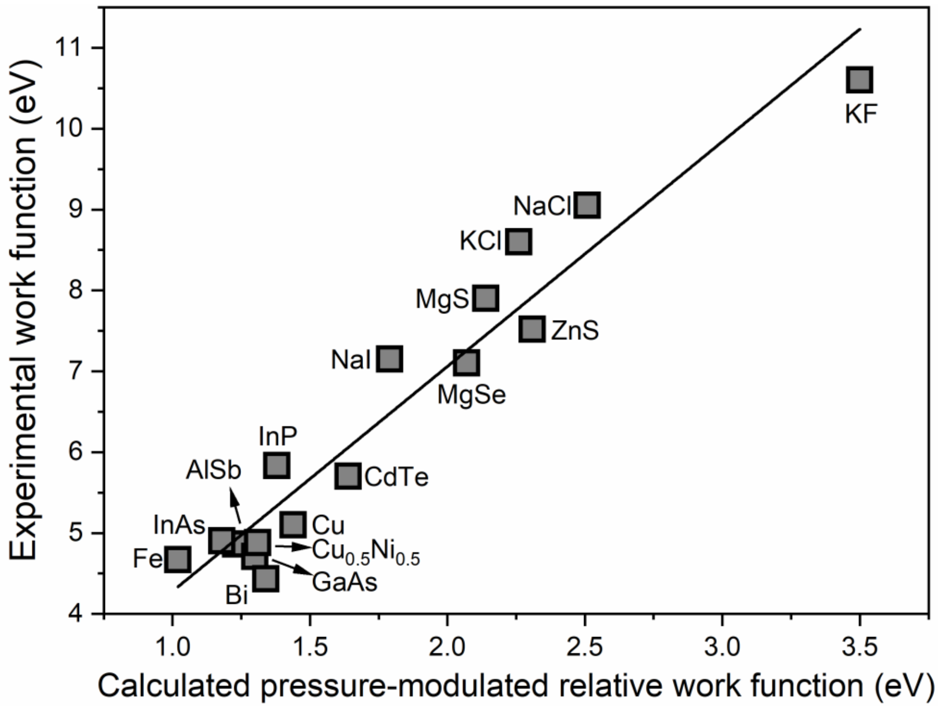

Extended Data Fig. 4 Work function calculation of some compounds for the comparison of their experimental data[1-5]. The squared Pearson correlation coefficient excels 0.93 of linear fitting.

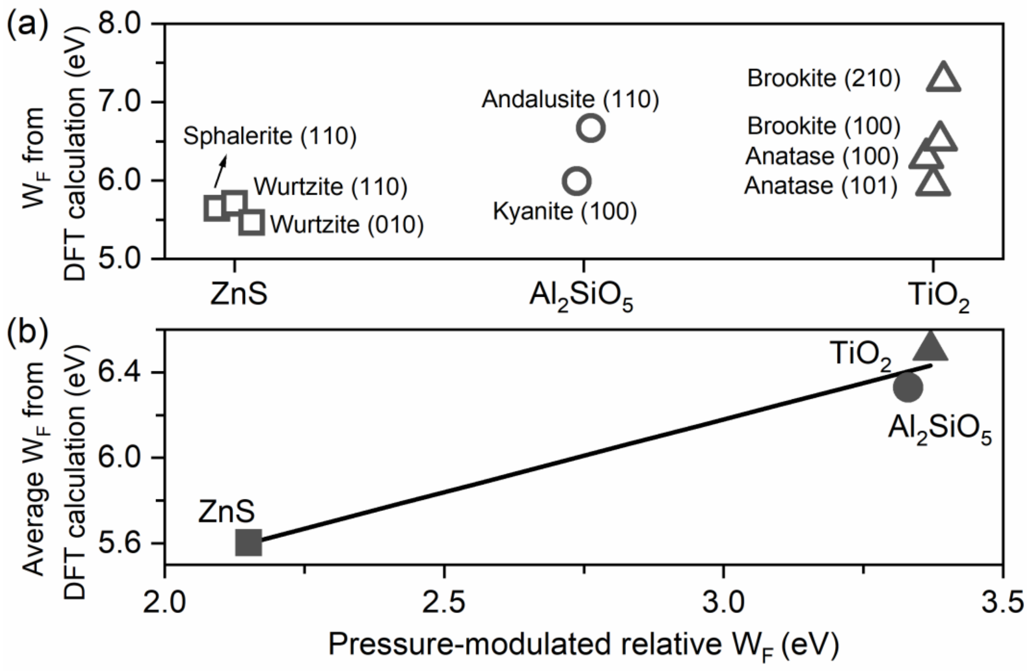

Extended Data Fig. 5 The comparison of the predicted work function (W_F) and calculated one. (a) The work function of some typical minerals with polymorphism at zero pressure, obtained from density functional theory (DFT) calculations. The selected surface corresponds to the most referential crystal plane of that mineral with the lowest energy. (b) The comparison between our used pressure-modulated relative work function values and the calculated work function average values from DFT. The squared Pearson correlation coefficient of linear fitting excels 0.97.

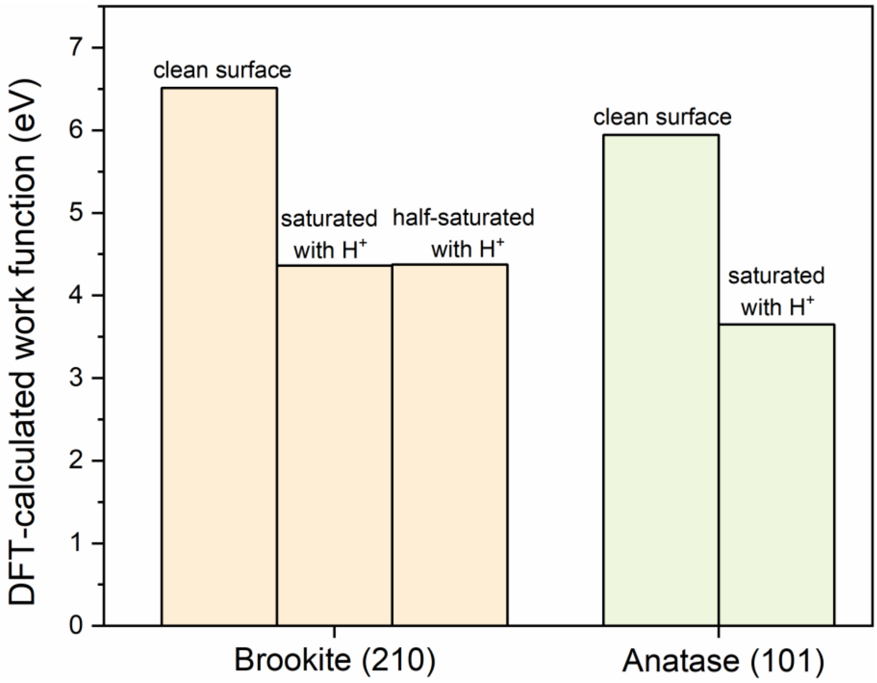

Extended Data Fig. 6 DFT-based calculation of work function for brookite and anatase minerals with clear surface or surface saturated with H^+ species.

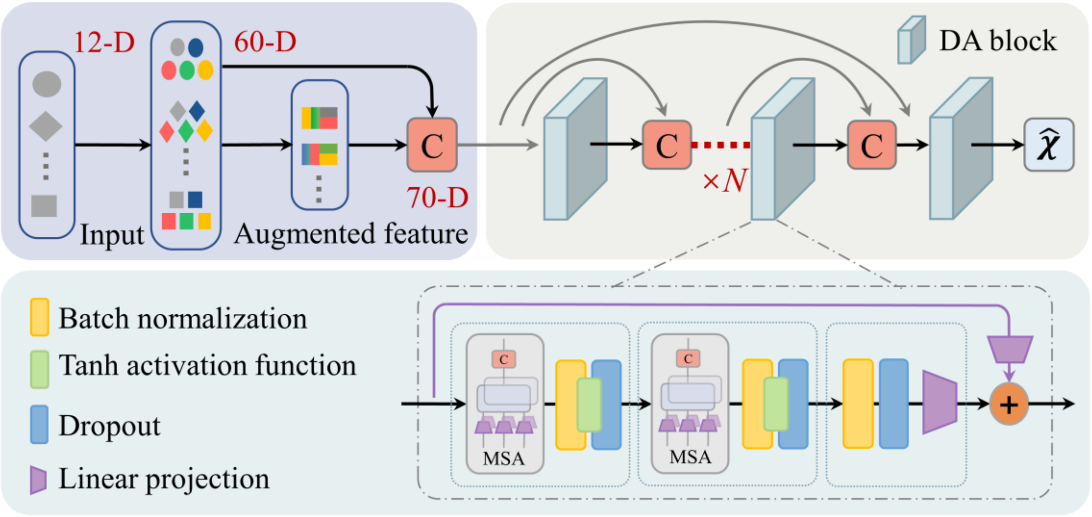

Extended Data Fig. 7 Schematic diagram of feature augmentation (the top-left component) and Dense Attention Network (DAN) (the top-right component) in deep learning model to acquire predicted element electronegativity ($\hat{\chi}$). Augmented-descriptor is generated using operation augmentation and symbolic transformer methods, increasing the dimensionality of features from initial 12 to 70. A deep learning model Dense Attention Network (DAN) fits the data. DAN consists of densely-connected DA blocks, which includes Multi-head self-attention module (MSA), Batch Normalization, Dropout, Linear Projection and Tanh activation function.

Extended data Table 1. The element descriptors used to numerically represent an element to train electronegativity model.

Category	Descriptors	Count
Electronic structure	Number of *s, p, d, f* valence electrons	4
Physical property	Ionization energy (ref.),<br>Atomic weight (ref.),<br>Pauling electronegativity (ref.)	3
Periodic table location	Row, group, pseudo row (ref.), atomic number	4

Extended data Table 2. Dense Attention Network hyperparameters settings.

Hyperparameters		Value
DAN Architecture	Number of DA blocks	3
	Number of MSA heads	8
	Dimension of MSA query, key, value tensor	18
	Probability of Dropout	0.5
	ABTD-1 output dimension.	65
	ABTD-2 output dimension	35
	BDL output dimension	1
Data augmentation	Feature gaussian noise μ	0
	Feature gaussian noise σ	0.01
	Label gaussian noise μ	0
	Label gaussian noise σ	0.002
Training process	Number of training epochs	750
	The number of k in k-fold bagging	5
	Batch size	64
	Learning rate	0.0019

Extended data Table 3. Comparison of the performance on the element electronegativity testing set. For the fairness of comparison, all methods use 5-fold bagging ensemble learning technology.

Method	RMSE	MAE	R^2
Ours	0.425	0.283	0.987
TabNet	0.969	0.817	0.943
NeuralNetFastAI	0.624	0.457	0.972
CatBoost	0.556	0.379	0.978
XGBoost	0.590	0.461	0.969
LightGBM	0.521	0.363	0.979
Random Forest	0.620	0.430	0.973

Extended data Table 4. The impact of different training strategies.

Settings	RMSE	MAE	R^2
Baseline	1.013	0.752	0.947
+ Operation augmentation	0.589	0.456	0.977
+ Symbolic transformer	0.570	0.434	0.977
+ MSA	0.559	0.465	0.979
+ Dense connection	0.529	0.370	0.980
+ 5-fold bagging	0.425	0.283	0.987

Supplementary References:
1. Fischer, T. E. Reflectivity, photoelectric emission, and work function of AlSb. *Physical Review* **139**, A1228 (1965).
2. Gobeli, G. W. & Allen, F. G. Photoelectric properties of cleaved GaAs, GaSb, InAs, and InSb surfaces; comparison with Si and Ge. *Physical Review* **137**, A245 (1965).
3. Work function values of InP, MgS, ZnS, MgSe, KCl, NaCl, NaI, KF and $Cu_{0.5}Ni_{0.5}$ come from https://materials.springer.com/.
4. Kapadnis, R. S. *et al*. Cadmium telluride/cadmium sulfide thin films solar cells: a review. *ES Energy & Environment* **10**, 3-12 (2020).
5. Haynes, W. M. *CRC Handbook of Chemistry and Physics 95nd edition* (CRC Press/Taylor and Francis, Boca Raton, 2014).
